# Capsulized Fecal Microbiota Transplantation Induces Remission in Patients with Ulcerative Colitis by Gut Microbial Colonization and Metabolite Regulation

**DOI:** 10.1128/spectrum.04152-22

**Published:** 2023-04-24

**Authors:** Qiongyun Chen, Yanyun Fan, Bangzhou Zhang, Changsheng Yan, Qiang Zhang, Yuhao Ke, Zhangran Chen, Lin Wang, Huaxiu Shi, Yiqun Hu, Qingwen Huang, Jingling Su, Chenxi Xie, Xu Zhang, Lixiang Zhou, Jianlin Ren, Hongzhi Xu

**Affiliations:** a Department of Gastroenterology, Zhongshan Hospital of Xiamen University, School of Medicine, Xiamen University, Xiamen, China; b Institute for Microbial Ecology, School of Medicine, Xiamen University, Xiamen, China; c Xiamen Key Laboratory of Intestinal Microbiome and Human Health, Zhongshan Hospital of Xiamen University, Xiamen, China; d Department of Digestive Disease, School of Medicine, Xiamen University, Xiamen, China; Yangzhou University

**Keywords:** fecal microbiota transplantation, ulcerative colitis, capsules, microbiome, gut microbiome, metabolism

## Abstract

Fecal microbiota transplantation (FMT) can induce clinical remission in ulcerative colitis (UC) patients. Enemas, nasoduodenal tubes, and colonoscopies are the most common routes for FMT administration. However, there is a lack of definitive evidence regarding the effectiveness of capsulized FMT treatment in UC patients. In this study, we administered capsulized FMT to 22 patients with active UC to assess the efficiency of capsulized FMT and determine the specific bacteria and metabolite factors associated with the response to clinical remission. Our results showed that the use of capsulized FMT was successful in the treatment of UC patients. Capsulized FMT induced clinical remission and clinical response in 57.1% (12 of 21) and 76.2% (16 of 21) of UC patients, respectively. Gut bacterial richness was increased after FMT in patients who achieved remission. Patients in remission after FMT exhibited enrichment of *Alistipes* sp. and Odoribacter splanchnicus, along with increased levels of indolelactic acid. Patients who did not achieve remission exhibited enrichment of Escherichia coli and Klebsiella and increased levels of biosynthesis of 12,13-DiHOME (12,13-dihydroxy-9Z-octadecenoic acid) and lipopolysaccharides. Furthermore, we identified a relationship between specific bacteria and metabolites and the induction of remission in patients. These findings may provide new insights into FMT in UC treatment and provide reference information about therapeutic microbial manipulation of FMT to enhance its effects. (This study has been registered at ClinicalTrails.gov under registration no. NCT03426683).

**IMPORTANCE** Fecal microbiota transplantation has been successfully used in patients. Recently, capsulized FMT was reported to induce a response in patients with UC. However, limited patients were enrolled in such studies, and the functional factors of capsulized FMT have not been reported in the remission of patients with UC. In this study, we prospectively recruited patients with UC to receive capsulized FMT. First, we found that capsulized FMT could induce clinical remission in 57.1% of patients and clinical response in 76.2% after 12 weeks, which was more acceptable. Second, we found a relationship between the decrease of opportunistic pathogen and lipopolysaccharide synthesis in patients in remission after capsulized FMT. We also identified an association between specific bacteria and metabolites and remission induction in patients after capsulized FMT. These findings put forward a possibility for patients to receive FMT at home and provide reference information about therapeutic microbial manipulation of FMT to enhance its effects.

## INTRODUCTION

Inflammatory bowel disease (IBD), mainly defined as either ulcerative colitis (UC) or Crohn’s disease (CD), is a chronic and relapsing intestinal inflammation involving various factors and an abnormal immune response (1). Ulcerative colitis is a continuous inflammation of the colonic mucosa that usually affects the rectum and gradually spreads to the entire colon. The inflammation caused by an abnormal immune response of the intestinal mucosa plays an important role in the pathogenesis of UC. The therapy-related options include 5-aminosalicylic acid (5-ASA), corticosteroids, and immunosuppressive agents. These drugs result in a periodic remission and are likely to result in a relapse (2). Therefore, it is necessary to explore new and effective treatments for UC. Because of the pathophysiological role of microbes in IBD, an alternative therapeutic approach involving the modulation of microbiota may be effective (3, 4).

For decades, fecal microbiota transplantation (FMT), the administration of a fecal suspension obtained from healthy donors into the gastrointestinal tract of a patient, has been used successfully for bacteriotherapy in individuals with Clostridioides difficile infections (CDIs) (5). This has encouraged studies that examine the use of FMT as a potential therapy for other diseases possibly influenced by the microbiome. An increasing number of subjects have recently undergone FMT as a treatment for IBD, especially UC. FMT has demonstrated variable efficacy against active UC. It appears to be more effective than other treatment methods involving antibiotics, probiotics, and prebiotics (4) and is associated with improved gastrointestinal symptoms, reduced diarrhea, and gut microbiota-related alterations (6–8). The outcomes of FMT treatment in UC patients were highly heterogeneous in various centers and were different from the stable response to FMT treatment in individuals with CDI. In a large multicenter, randomized, double-blind clinical trial in Australia, steroid-free clinical remission was achieved in 32% (12 of 38) of participants receiving low-density donor FMT prepared anaerobically after 1 week of treatment (6). Another clinical trial involving UC patients reported steroid-free clinical remission in 18 of 41 (44%) patients who underwent colonoscopic infusions once and received enemas 5 days per week for 8 weeks (9). There is no doubt that the high-quality handling of donor stool, patients who fulfil certain criteria, and the use of certain transfusion methods are essential for successful FMT treatment. Although the efficacy of FMT in UC has been assessed in numerous randomized controlled trials, more extensive clinical studies are required to determine its efficacy conclusively.

Currently, multiple modes of FMT administration, including colonoscopy, nasoduodenal tubes, enemas, and capsules, are available to clinicians (10–14). Among these, invasive operations are inconvenient and increase the risk of adverse events and medical costs, especially in individuals frequently undergoing FMTs. Hence, most patients reject FMT treatment, despite the fact that it may result in good outcomes. Therefore, the search for a convenient, efficient, and effective FMT method has practical significance in applications in clinical practice. A systematic review and meta-analysis of the CDI cure rate data showed that FMT, when performed with colonoscopy, was superior to an enema and comparable to treatment with oral capsules (15). Besides, there are several reports on capsulized FMT in patients with UC (10, 13, 16). In these studies, capsulized FMT induced a clinical response in UC patients and improved patients' Mayo scores or endoscopically confirmed mucosal healing. However, limited numbers of patients (fewer than 10) were enrolled in such studies. Furthermore, the functional factors of capsulized FMT have not been reported in the remission of patients with UC. A trial should be conducted on a larger scale to explore the advantage of capsulized FMT and determine its impact on the clinical outcomes of patients.

Here, we investigated the longitudinal dynamics of serum metabolites and the gut microbiome of bacteria in UC patients after capsulized FMT treatment. We aimed to determine the efficiency of treatment of UC patients with capsulized FMT and determine the functional factors associated with clinical remission through multiomic analyses.

## RESULTS

### Beneficial outcomes of FMT.

**FIG 1:**
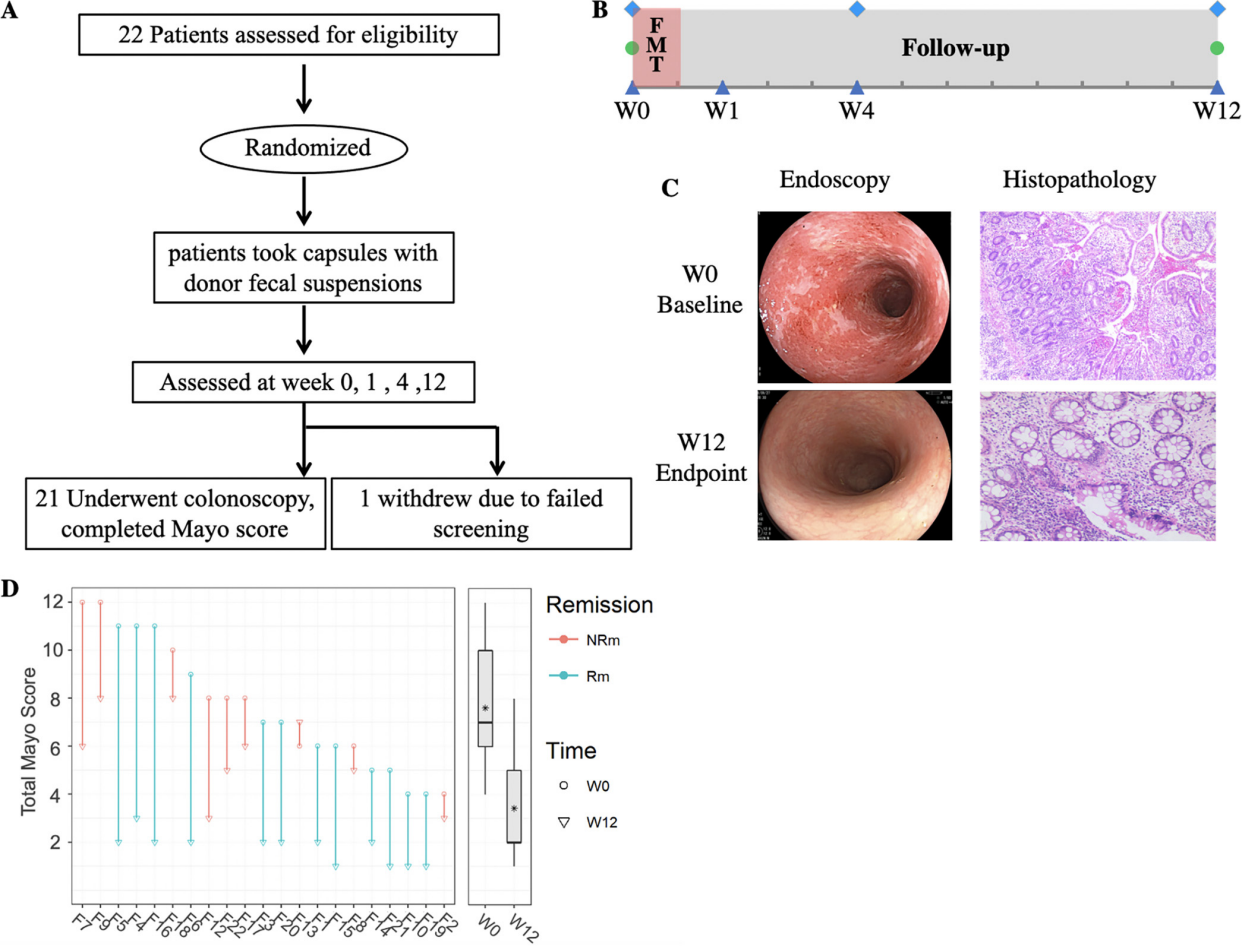
Effect of fecal microbiota transplantation on 12-week remission in patients with ulcerative colitis. (A) Trial profile of fecal microbiota transplantation in patients with ulcerative colitis; (B) time points for obtaining fecal samples (triangles), clinical evaluation by Mayo scoring (diamonds), and colonoscopy (circles) during this clinical trial. Shown are changes in endoscopy and hematoxylin-eosin staining (C) and Mayo scores (D) for UC patients before and after treatment with FMT. The parallel line shows the change in Mayo score for individual patients, with a solid line indicative of values for FMT. Each line started at the baseline (W0 [circle]) and finished at its endpoint (W12 [triangle]). The achievement of clinical remission is indicated in green, and nonclinical remission is shown in red. Asterisks in in box plots represent the mean Mayo scores of each group. The significance was determined by paired Wilcoxon rank sum test. FMT, oral capsulized fecal microbiota transplantation.

Between June 2016 and June 2019, 22 patients with active UC were recruited and assessed for eligibility. Enrolled patients received capsulized FMT treatment and completed the final follow-up at week 12. During the follow-up visits, one patient failed the screening process and was thus excluded. The clinical trial protocol is shown in Fig. 1A and B. Baseline characteristics of patients and measurements of disease activity are shown in Table 1. The mean age of enrolled patients was 42.62 years. Three patients were female, and the mean total Mayo score was 7.62 at the baseline. Among these, 17 patients received mesalazine (2.0 g twice a day [b.i.d.]), and 4 patients were glucocorticoid dependent.

**TABLE 1:** Baseline characteristics of patients with UC and the outcomes after treatment by FMT

Characteristic^*a*^	Result for UC patients (*n* = 22)^*b*^
Sex, no. (%)	
Women	3 (13.6)
Men	19 (86.4)
Age, yr, mean (range)	42.5 (20–68)
Left-sided disease only, no. (%)	7 (31.8)
Total Mayo score	7.62 (4–12)
Inflammatory markers, mean (range)	
CRP, mg/L	5.03 (0.15–64.89)
ESR, mm/h	14.42 (0.2–30.7)
WBC count, 10 × 9/L	7.7 (3.62–18.12)
Neutrophil count, 10 × 9/L	4.96 (1.48–14.13)
Neutrophil ratio, % (range)	60.09 (38.3–75.3)
Outcomes, no. (%)	
Clinical response	16 (76.2)
Clinical remission	12 (57.1)
Endoscopic remission	10 (47.6)

a CRP, C-reactive protein; ESR, erythrocyte sedimentation rate; WBC count, white blood cell count.

b The efficacy analysis included 21 patients, excluding 1 patient who lost communication during the study.

Over the 12-week study, a significant reduction in the total Mayo score was observed compared to the baseline. The patients who achieved a Mayo score with a ≥3-point reduction and a reduction of ≥30% at week 12 were considered responders (Rps). The patients who achieved a Mayo score of ≤2 at week 12 were considered to be in clinical remission (Rm). At the baseline, there were no significant differences in the total Mayo score between responders (Rps) and nonresponders (NRps) or patients in the remission (Rm) and nonremission (NRm) stages. The mean total Mayo score significantly (*P* < 0.001) decreased after capsulized FMT treatment at week 12 (Fig. 1D). After FMT treatment, the intestinal mucosal appeared to be repaired, and the outcomes associated with ulcers or healthy mucosa were improved. The results of the histopathological examination showed that the mucosal glands became regular, the numbers of acute and chronic inflammatory cells decreased significantly, compared to the numbers observed previously, and no cryptitis or crypt abscesses were observed in the mucosa (Fig. 1C). The primary endpoint of clinical remission was achieved in 12 (57.1%) out of 21 patients with UC after capsulized FMT administration: of these, 10 (47.6%) patients achieved endoscopic remission (Table 1). Clinical response was observed in up to 76.2% (16/21) of UC patients (see Fig. S1 in the supplemental material). Notably, some patients who received capsulized FMT could get relief from clinical symptoms at week 4. Four patients who received glucocorticoids were successfully treated with mandatorily tapered doses during the follow-up.

Satisfactorily, no serious adverse events occurred during the study period, and capsulized FMT treatment was well tolerated. The most frequently occurring adverse events were gastrointestinal complaints, including increased bowel movements (*n* = 2 [9.15%]), stool with capsule shells (*n* = 1 [4.5%]), and abdominal pain (*n* = 1 [4.5%]). These adverse events were mild, self-limiting in 24 h, and usually occurred after the first FMT treatment was administered (Table 2). No signs of infection or changes in the number of white blood cells were observed after capsulized FMT treatment (Table 3).

**TABLE 2:** Adverse events during the 12-week study

AE^*a*^	Result for enrolled UC patients (*n* = 22)^*b*^
Total AEs, *n*	6
Total SAEs, *n* (%)	0 (0)
Withdrawal due to FMT-related AE, *n* (%)	1 (4.5)
Abdominal pain, *n* (%)	2 (9.1)
Loose stools, *n* (%)	2 (9.1)
Vomiting, *n* (%)	0 (0)
Fecal with capsules, *n* (%)	1 (4.5)

a AE, adverse event; SAEs, serious adverse events.

b Data are number of events or number of patients with percentage shown in parentheses.

**TABLE 3:** Laboratory tests of UC patients during FMT treatment^*a*^

Parameter	Rm group				NRm group			
	Baseline	W1	W4	W12	Baseline	W1	W4	W12
CRP, mg/L, mean (range)	6.82 (0.15–64.89)	1.45 (0.3–2.86)	1.02 (0.21–2.19)	2.83 (0.34–17.66)	3.41 (0.4–9.67)	1.63 (0.37–8.4)	11.56 (0.52–54.2)	13.29 (0.62–38.5)
ESR, mm/h, mean (range)	15.33 (4.2–35.0)	13.49 (3.2–42.7)	9.71 (1.7–21.8)	7.1 (1.0–12.7)	11.83 (0.2–18.3)	12.69 (0.1–21.5)	15.51 (0.26–48.5)	12.48 (0.1–23.6)
WBC count, 10 × 9/L, mean (range)	7.63 (3.86–12.12)	5.79 (4.98–7.1)	6.79 (4.39–10.32)	6.53 (4.0–9.1)	7.81 (3.62–18.12)	9.06 (4.7–16.74)	5.64 (2.62–8.55)	6.53 (4.73–7.65)
Monocyte count, 10 × 9/L, mean (range)	0.50 (0.27–0.72)	0.412 (0.2–0.66)	0.43 (0.28–0.67)	0.41 (0.24–0.73)	0.51 (0.26–1.2)	0.72 (0.42–0.95)	0.46 (0.21–0.66)	0.41 (0.35–0.48)
Neutrophil count, 10 × 9/L, mean (range)	4.9 (1.48–8.14)	3.53 (1.36–5.75)	4.3 (2.24–6.72)	4.41 (2.73–6.46)	5.03 (2.24–14.13)	6.85 (2.83–13.1)	3.36 (1.33–6.98)	3.33 (2.54–4.84)

a Rm group, patients in clinical remission; NRm group, patients in the nonremission stage; W1, W4, and W12, weeks 1, 4, and 12, respectively; CRP, C-reactive protein; ESR, erythrocyte sedimentation rate; WBC count, white blood cell count.

### Alterations in gut microbiota after FMT.

Bacterial α diversity was assessed by determining richness (observed and Chao1), Shannon diversity, and Pielou’s evenness based on 16S V3-V4 sequencing. As expected, the bacterial α diversity, observed richness (*P* < 0.05), and Shannon (*P* < 0.05) indices were significantly lower in UC patients than in healthy individuals (Fig. S2A). The analysis of β diversity via principal-component analysis (PCA) revealed that the gut bacterial communities of patients with UC were clearly (permutational multivariate analysis of variance [PERMANOVA]; *F* = 4.131; *P* < 0.001) clustered separately from the healthy group (Fig. S2B). This pattern was significantly associated with a higher abundance of Escherichia*_Shigella*, *Veillonella*, Enterobacter, and *Collinsella*, and a lower abundance of *Alistipes* and *Akkermansia* in UC patients (Fig. S2B and C).

**FIG 2:**
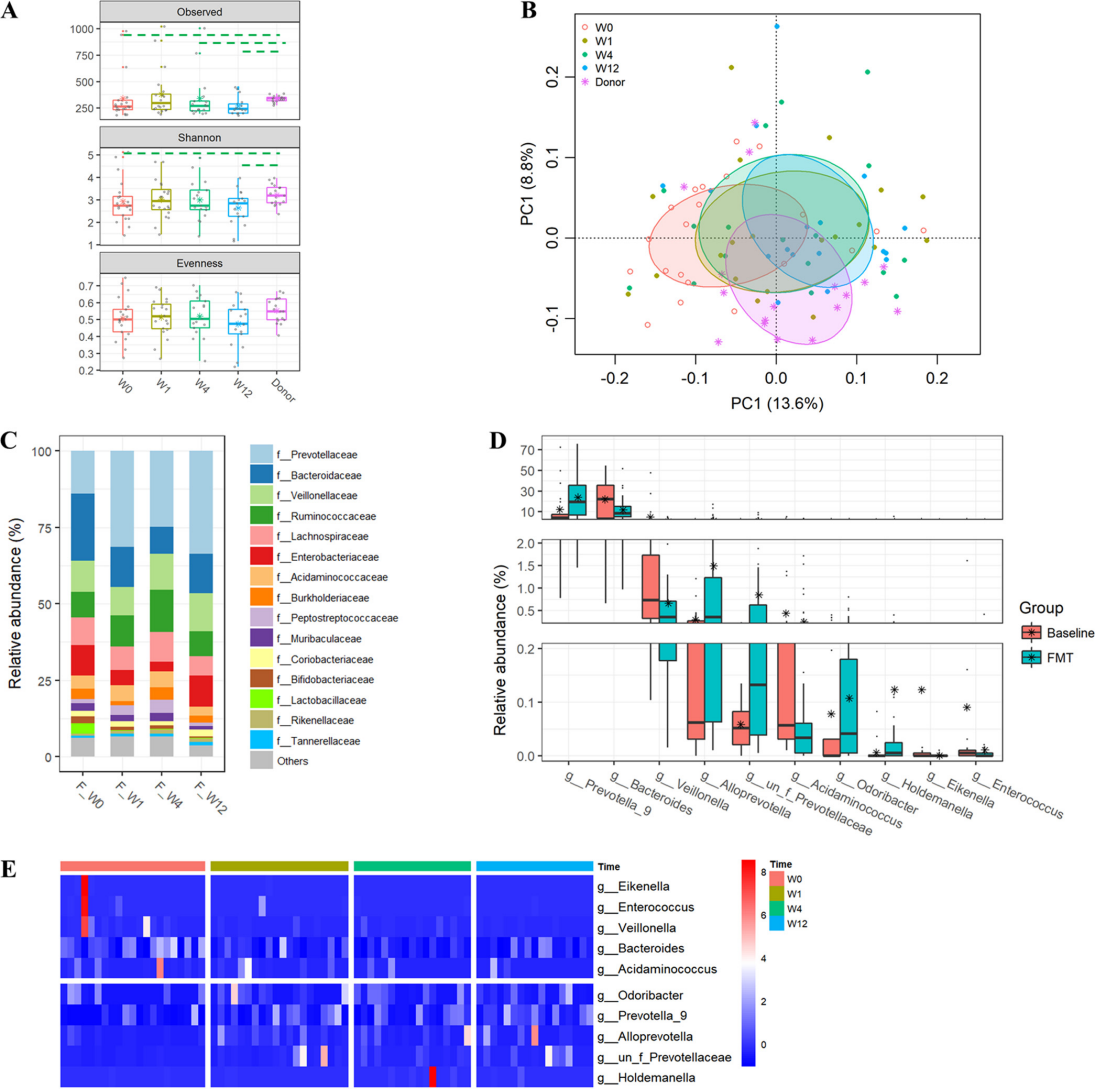
Alterations in gut bacterial communities of UC patients after FMT. (A) Estimating the α diversity by the richness (observed OTUs and Chao1) and Shannon and Pielou’s evenness. The dashed line indicates significant (*P* < 0.05) differences between the two groups covered by the line. (B) Differences in gut bacterial community structures among UC patients at the baseline, at other time points during follow-up visits, and donors assessed by principal-component analysis of the Euclidean distance of OTU abundances. Data were subjected to a Hellinger transformation. The top 10 genera were fitted to PCA with a significance of *P* < 0.05. (C) Relative abundances of the top 15 families in samples obtained from patients in the FMT group. (D) Relative abundances of genera were significantly different between samples at the baseline and after FMT treatment. (E) Heat map of the genera showing significant differences at different times in the FMT group, based on the Z-score-transformed relative abundance of genera at all time points.

Similarly, both richness indices were significantly higher in donor samples than in baseline samples of patients. The bacterial richness of samples from UC patients increased significantly after capsulized FMT and was comparable to that of samples obtained from donors at week 1 but was reduced at weeks 4 and 12 (Fig. 2A). Shannon diversity also increased at weeks 1 and 4 after capsulized FMT. Principal-component analysis revealed notable shifts (PERMANOVA; *F* = 1.7913; *P* < 0.001) in overall microbial communities of patients undergoing FMT toward the cluster observed in donor samples (Fig. 2B). Notably, *Prevotellaceae* was observed to become dominant at the family level in the profiles of patients receiving FMT (Fig. 2C). In addition, enrichment of *Prevotella_9* and depletion of *Bacteroides* and Escherichia*_Shigella* were observed in the top 25 genera (Fig. S3). In comparison with the baseline, levels of *Prevotella_9*, *Alloprevotella*, and *Odoribacter* were significantly increased in samples obtained from UC patients after FMT, while those of *Bacteroides*, *Veillonella*, and *Enterococcus* were significantly decreased (Fig. 2D and E). The shifts in microbial profiles in stool samples obtained from patients after FMT were more remarkable at the operational taxonomic unit (OTU) level (Fig. S4).

### Enrichment of beneficial bacteria associated with the clinical remission after FMT accompanied the decrease in levels of opportunistic pathogens.

**FIG 3:**
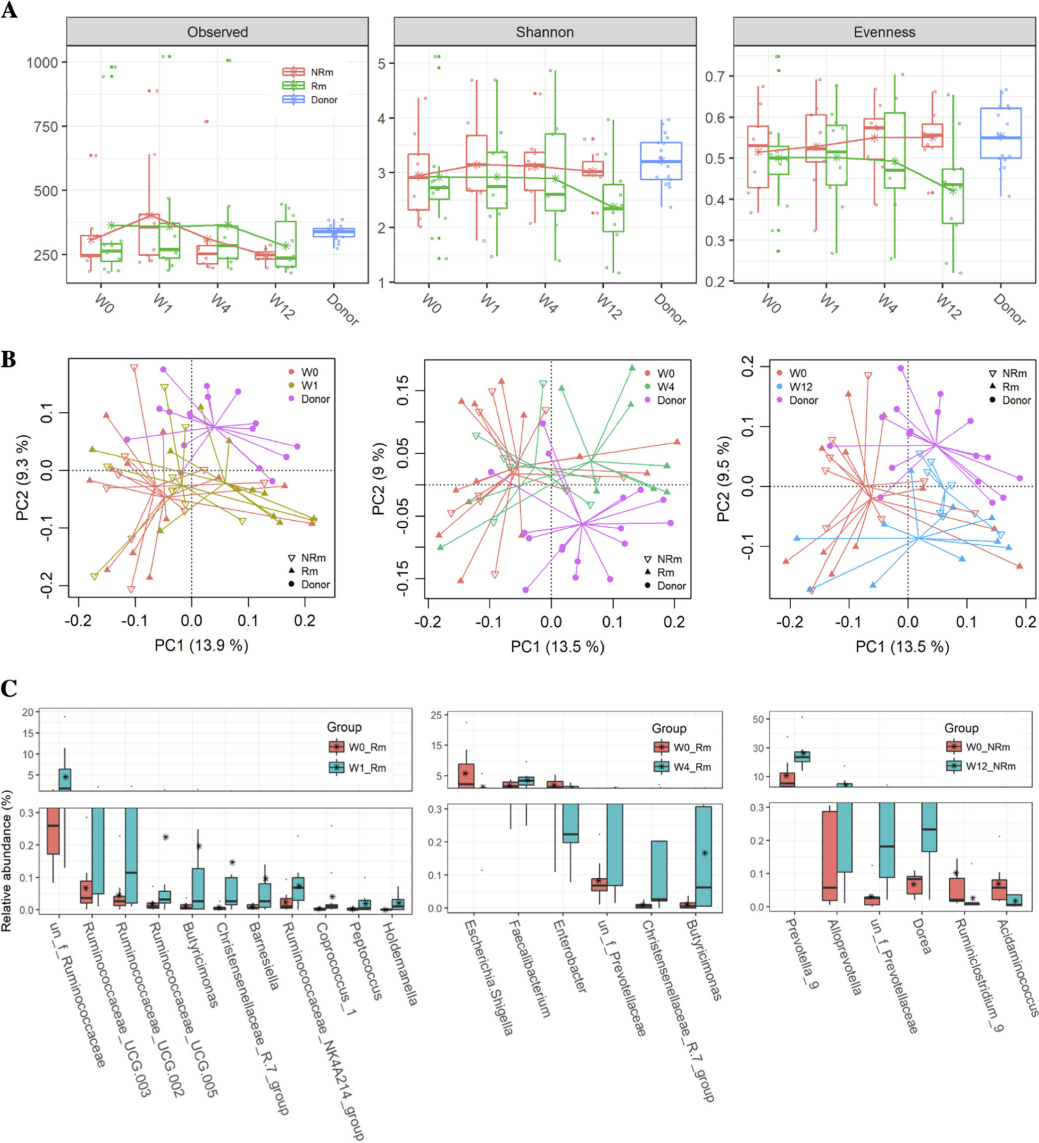
Changes in gut bacterial communities associated with Rm and NRm after FMT. (A) α diversity of Rm, NRm, and donor samples, estimated by richness (observed OTUs) and Shannon and Pielou’s evenness. The dashed line indicated significant (*P* < 0.05) differences between the two groups covered by the line. (B) Differences in gut bacterial community structures of Rm, NRm, and donors between the baseline and other time points during follow-up visits after FMT were assessed by PCA based on the Euclidean distance of OTU abundance. Data were subjected to a Hellinger transformation. (C) The relative abundance of the bacterial genera was uniquely associated with clinical remission. The genera were identified by analyzing significantly different genera between the baseline and other time points in Rm using the paired rank sum test and then removing the genera that were simultaneously significantly different between the baseline and other time points in NRm (W1 and W4) and vice versa to determine the right insert (W12).

UC patients who achieved clinical remission exhibited efficient and durable changes in their gut microbiota after FMT. Based on 16S V3-V4 sequencing, gut bacterial richness was increased in the clinical remission (Rm) group and was comparable to that of donors during the follow-up visits after FMT. In contrast, the richness indices at week 4 and week 12 after FMT were significantly (*P* < 0.05) lower than those of donors in the nonremission group (NRm) group. However, no significant differences were observed between the Rm and NRm groups at each time point (Fig. 3A). The trend of increase in α diversity after FMT was more remarkable in data sets sequenced using metagenomic techniques (Fig. S5). Interestingly, the Shannon and evenness values were marginally (0.05 < *P* < 0.1) lower, while the richness was relatively higher in the Rm group than in the NRm group at week 12, which implies that some taxa had been grafted effectively during FMT and maintained their growth advantage in the Rm group. Overall, gut bacterial communities in stool samples obtained from patients who achieved remission were clustered closer to those in stool samples obtained from donors 1 week after FMT, whereas bacterial communities in stool samples obtained from NRm patients were not closer to those in stool samples obtained from donors until week 12 (Fig. 3B; Fig. S5). Compared with the baseline, significant changes in bacterial communities were detected at both week 1 (PERMANOVA; *F* = 1.7603; *P* = 0.0384) and week 4 (PERMANOVA; *F* = 1.926; *P* = 0.034) in the Rm group, but not in the NRm group in both 16S rRNA and metagenomic data sets (see Table S1 in the supplemental material). More importantly, there were significant differences between the Rm and NRm groups at week 4 (Fig. 3B; Table S1). These results indicated that it could be important to evaluate the microbial changes associated with remission at 4 weeks after FMT.

To identify the taxa associated with clinical remission, the differences in genera in the Rm or NRm groups were evaluated between each time point after FMT, and the baseline values were analyzed by the paired rank sum test. *Butyricimonas* and *Ruminococcacae* were exclusively increased at week 1 after FMT in the Rm group (not in the NRm group). Notably, levels of well-known beneficial bacteria (*Faecalibacter* and *Butyricimonas*) were exclusively enriched, and those of opportunistic pathogens (Escherichia*_Shigella* and Enterobacter) were exclusively decreased at week 4 in the Rm group alone (Fig. 3C). *Prevotella_9* and *Alloprevotella* were specifically increased in the NRm group at week 12. These findings indicated that clinical remission after FMT was associated with the eradication of opportunistic pathogens and the enrichment of beneficial bacteria. Besides, we found that the relative abundance of members of *Ruminococcus_gnavus_group* was significantly higher in the NRm group than in the Rm group at baseline, week 1, and week 4 after FMT (Fig. S6). *Pedioccus*, *Enterococcus*, and *Motganella* showed similar trends (Fig. S6). These taxa might be potential biomarkers that could predict clinical outcomes before FMT.

### Shotgun metagenomic analyses confirmed the signatures associated with clinical remission.

**FIG 4:**
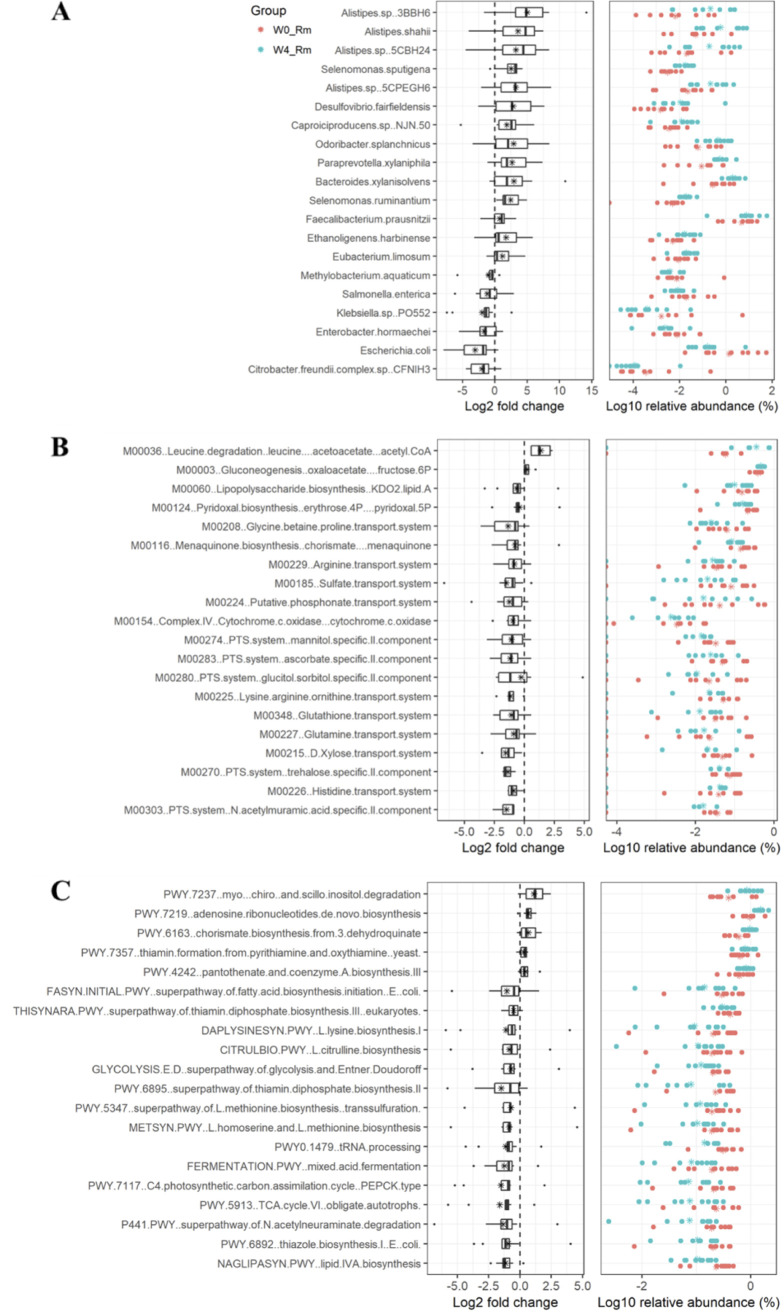
Microbial signatures are specifically associated with clinical remission at week 4. Shown are (A) species, (B) KEGG modules, and (C) MetaCyc pathways whose abundance significantly decreased or increased uniquely in W4_Rm compared to the W0_Rm group (namely, not significantly differentiated in W4_NRm versus W0_NRm) during the shotgun metagenomic profiling of fecal samples. The significance was tested using the paired Wilcoxon test. The top 20 signatures of each category indicative of the relative abundance were visualized. Asterisks indicate the mean values of each signature.

**FIG 5:**
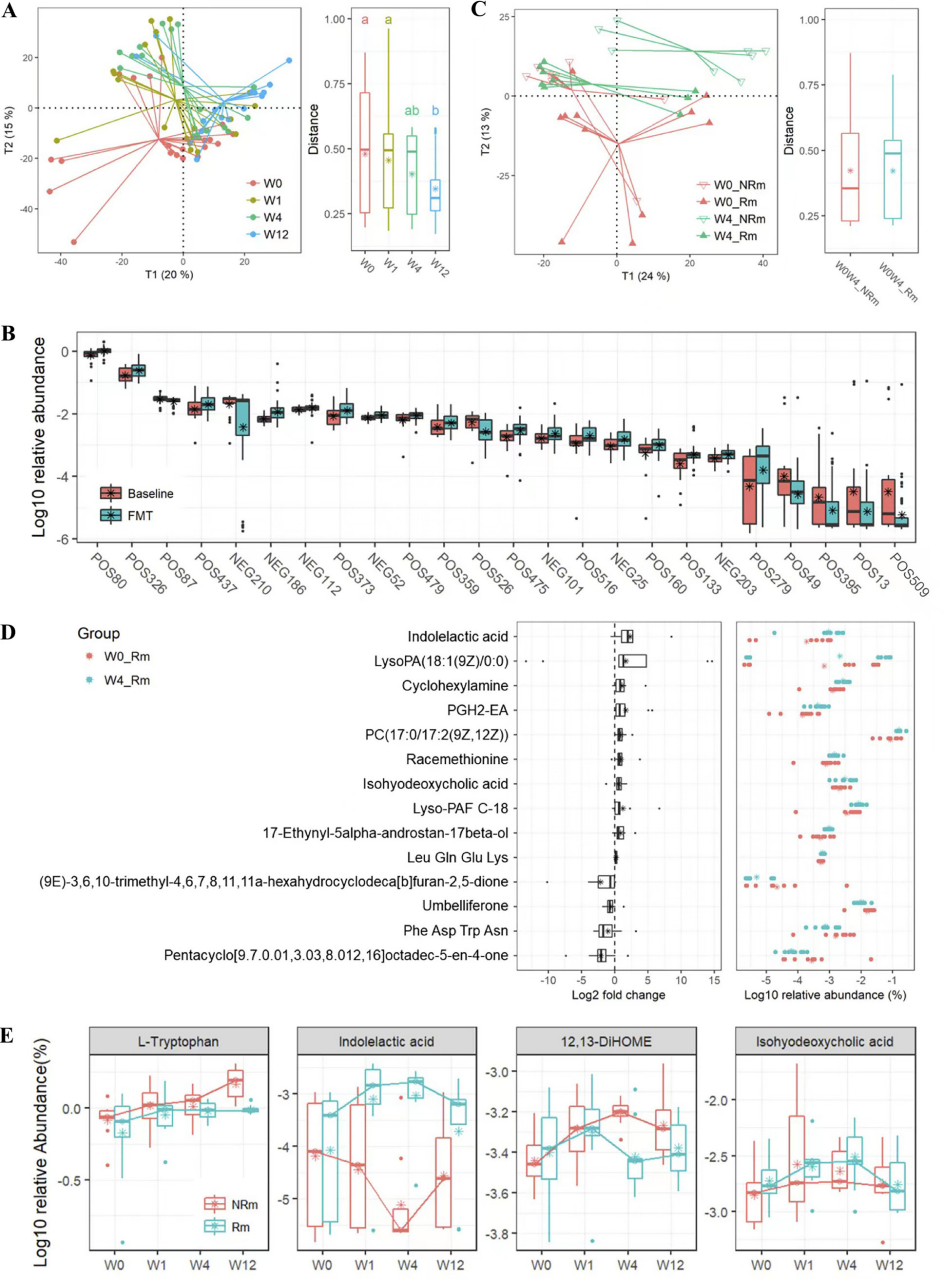
Metabolic profiles are associated with clinical remission at the baseline and time points after FMT. (A) PLS_DA of metabolomic profiles in the positive-ion model with regard to time points (left panel) and Euclidean distances of samples within the same time points (right panel). Data were subjected to a Hellinger transformation. Letters indicate groupings with significant differences. (B) Metabolites whose relative abundances were significantly different between the baseline and samples after FMT were filtered by variable importance if the variable importance projection (VIP) score was >1 and were structurally identified in databases. (C) PLS_DA of metabolomic profiles in the positive-ion model at weeks 0 and 4 after FMT after achieving clinical remission or not achieving clinical remission (left panel), as well as Euclidean distances of patients between week 0 and week 4 (right panel). Data were subjected to a Hellinger transformation. (D) Metabolites whose levels were significantly decreased or increased uniquely in W4_Rm, compared with the W0_Rm group (namely, not significantly differentiated in W4_NRm versus W0_NRm), were filtered using a VIP score of >1 and structurally identified in databases. (E) Relative abundance of partial metabolites that changed over time after FMT and were further stratified by clinical remission.

To further reveal the gut microbial signatures associated with clinical remission, the microbial data obtained after shotgun metagenomic analyses of week 0 Rm (W0_Rm) and W4_Rm and week 0 NRm (W0_NRm) and W4_NRm were analyzed using the paired Wilcoxon test. A total of 5,466 species were annotated using kraken2, of which the relative abundance of 937 species was significantly changed, specifically between W0_Rm and W4_Rm (Table S2). Among the top 20 species exhibiting a shift in abundance levels, the increased relative abundance of *Alistipes* (*Alistipes* sp. strains 3BBH6, 5CBH24, and 5CPEGH6 and Alistipes shahii), Odoribacter splanchnicus, and Faecalibacterium prausnitzii and decreased relative abundance of Escherichia coli, Enterobacter hormaechei, and Citrobacter freundii complex CFNIH3 were observed in the stool samples of patients who achieved clinical remission after FMT (Fig. 4A). In terms of functional microbial pathways, a total of 21 KEGG modules (Table S3) and 98 MetaCyc pathways (Table S4) were identified to be specifically associated with clinical remission. Acetoacetate plus acetyl coenzyme A (acetyl-CoA) (M00036) formation, gluconeogenesis to fructose-6P (M00003), adenosine ribonucleotide *de novo* biosynthesis (PWY-7219), and thiamine formation (PWY-7357) were uniquely increased in the Rm group at week 4. Lipopolysaccharide (LPS) biosynthesis-KDO2-lipid A (M00060), lipid IVA biosynthesis (NAGLIPASYN-PWY), and the superpathways for *N*-acetylneuraminate degradation (P441-PWY) were uniquely decreased, compared to those in the Rm group at week 0 (Fig. 5B and C). Taxa that mainly contributed to lipopolysaccharide biosynthesis pathways included Escherichia coli and Klebsiella (Table S5), and their abundance was also observed to be decreased in the W4_Rm group.

### Metabolomic profiling of UC patients achieving remission.

Global metabolomic analysis of serum samples revealed metabolic alterations and metabolites associated with clinical remission after capsulized FMT administration. As visualized in the partial least-squares discriminant analysis (PLS_DA) ordination, the metabolomic profiles of patients were shifted after FMT treatment in both positive- and negative-ion models (Fig. 5A; Fig. S7A). In particular, the metabolomic profiles of patients from the Rm and NRm groups showed considerable variations at week 4 after FMT (Fig. 5A). Overall, the 378 metabolites detected in the positive- or negative-ion models showed significant changes after FMT compared to the baseline (Table S6). Among these, 24 metabolites with a variable importance in projection (VIP) score of >1 were structurally identified in databases. These metabolites included l-tryptophan (POS80), 12,13-DiHOME (NEG203), and PGH2-EA (POS133) (Fig. 5B). There were significant differences in metabolomic profiles between week 0 and week 4 after FMT in both positive-ion (PERMANOVA; *F* = 2.375; *P* = 0.047) and negative-ion (PERMANOVA; *F* = 2.086; *P* = 0.041) models. In terms of clinical remission, metabolomic changes in patients from week 0 to week 4 in the Rm group were relatively more extensive than the changes from week 0 to week 4 in the NRm group (Fig. 5C; Fig. S7B). Specifically, significant differences (*P* < 0.05; VIP > 1) were observed during the structural identification of 14 metabolites between W0_Rm and W4_Rm (Fig. 5D), and these included increased levels of indolelactic acid, PGH2-EA, and isohyodeoxycholic acid. In contrast, we found a significant difference in the levels of 35 metabolites between W0_NRm and W4_NRm (*P* < 0.05; VIP > 1) (Fig. S8), including l-tryptophan, 12,13-DiHOME (12,13-dihydroxy-9Z-octadecenoic acid), and oleoyl ethyl amide. In addition, the metabolomic profile of W4_Rm was significantly different from that of W4_NRm (PERMANOVA; *F* = 3.171; *P* = 0.024). Notably, a significantly higher abundance of indolelactic acid was observed in W4_Rm compared to that in W4_NRm (Fig. 5E; Table S7), which was consistent with the differences between W4_Rm and W0_Rm. In comparison, a significantly lower relative abundance of 12,13-DiHOME was observed in W4_Rm compared to that in W4_NRm (Fig. 5E; Table S7) and in W0_NRm compared to that in W4_NRm (Fig. S8).

### Interomic correlations between gut microbes and metabolites.

**FIG 6:**
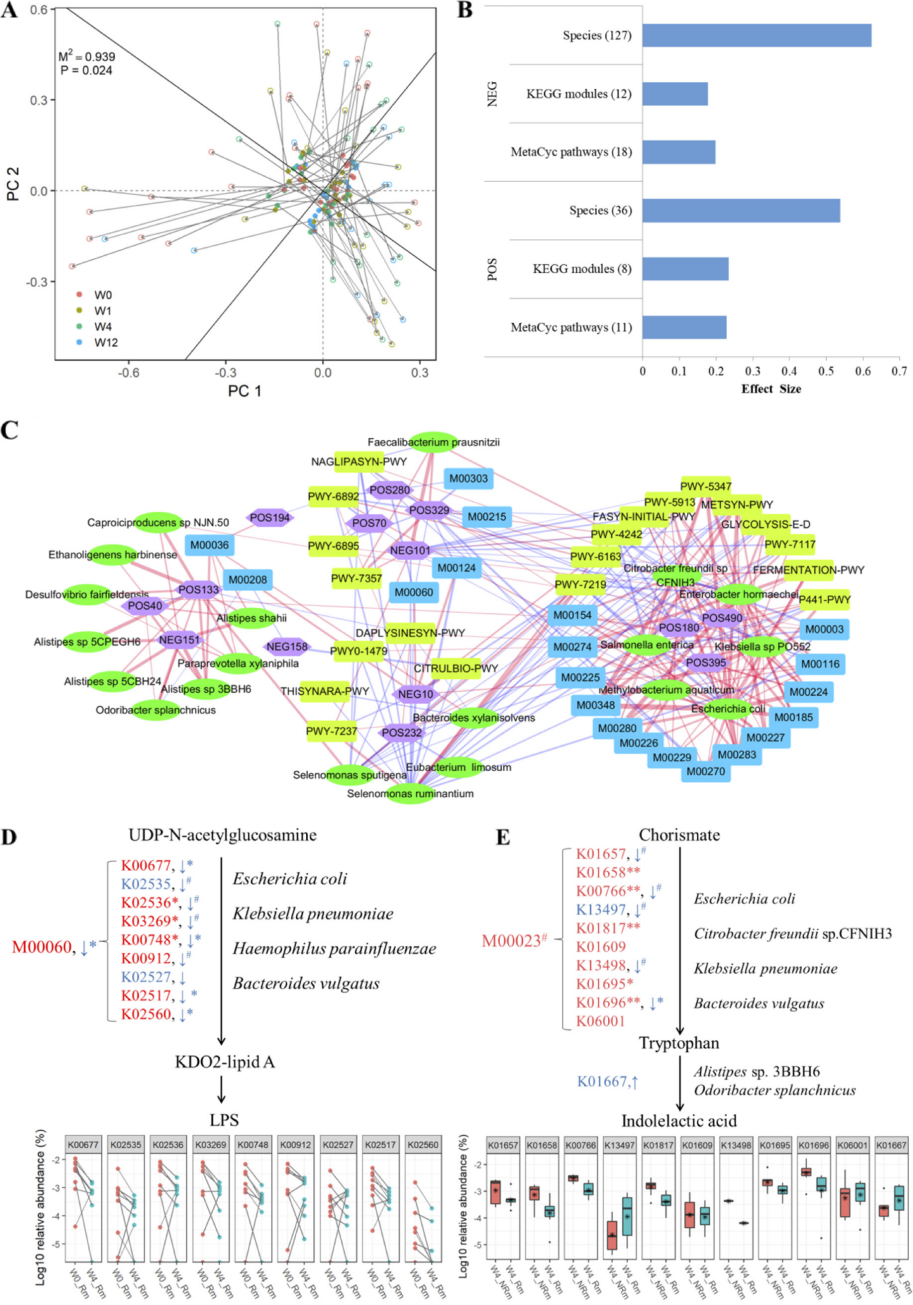
Interomic correlations between gut microbes and metabolites. (A) Procrustes analysis of gut microbial species and serum metabolites; (B) proportion of the total variations in serum metabolomes for different gut microbial species; (C) Pearson correlation-based network analysis of the top 20 gut microbial species, MetaCyc pathways, KEGG modules, and serum metabolites that were uniquely significantly different between W0_Rm and W4_Rm. Species with significant correlations (*P* < 0.05) were retained. (D) W4_Rm enriched and decreased gut microbiomes (species, genes, and pathways) associated with serum metabolites that contain genes involved in LPS biosynthesis (left panel) and tryptophan metabolism (right panel).

Procrustes analysis demonstrated the strong cooperative relationships between the gut microbiota and KEGG modules (M^2^ = 0.5005, *P* = 0.001) (Fig. S9A), MetaCyc pathways (M^2^ = 0.44; *P* = 0.001) (Fig. S9B), and serum metabolites (M^2^ = 0.939; *P* = 0.024) (Fig. 6A). Permutational multivariate analysis of variance (PERMANOVA)-based effect size analysis was performed to determine how each set of gut microbiomes affected metabolic profiles. The effect sizes of microbial species, MetaCyc pathways, and KEGG modules accounted for 53.7%, 22.3%, and 23.3% of metabolome variance in the positive model and 62.3%, 19.9%, and 17.8% of metabolome variance in the negative model, respectively (Fig. 6B). To determine the relationship between alterations in the gut microbiome and serum metabolism associated with clinical remission, Pearson correlation analysis was used to explore the differences between the top 20 gut microbial species (Fig. 4A), MetaCyc pathways (Fig. 4B), KEGG modules (Fig. 4C), and serum metabolites (Fig. 5D) that were significantly changed between W0_Rm and W4_Rm. A total of 256 significant (*P* < 0.05) shotgun metagenomic-based gut microbial features and metabolite correlations were identified (Fig. 6C), while only 10 significant correlations were identified between genera from the 16S V4 data set and metabolites (Fig. S10). Four major clusters of correlations were visualized via network analysis, of which one was characterized by increased levels of beneficial metabolites [POS133_PGH2-EA and NEG151_LysoPA(18:1(9Z)/0:0)] and microbial species (*Alistipes* sp., Odoribacter splanchnicus, etc.), while another was characterized by decreased levels of metabolites (POS490_Pentacyclo[9.7.0.01,3.03,8.012,16]octadec-5-en-4-one, POS180_Phe-Asp-Trp-Asn, etc.), opportunistic pathogens (Escherichia coli, Klebsiella sp. strain PO552, Salmonella enterica, etc.), and functional pathways (PWY-5347, the superpathway of l-methionine biosynthesis [transsulfuration]; FASYN-INITIAL-PWY, the superpathway of fatty acid biosynthesis initiation [E. coli]; M00348, the glutathione transport system; and M00227, the glutamine transport system). The correlation of positions between Faecalibacterium prausnitzii, NEG101_ Isohyodeoxycholic acid, and POS329_PC[17:0/17:2(9Z,12Z)] was observed in both metagenomic and 16S V4 data sets (Fig. 6C). Notably, we observed a significant decrease in the KEGG module M00060 and the pathway of KDO2-lipid A biosynthesis from UDP-*N*-acetylglucosamine to LPS in patients achieving remission at week 4 (W4_Rm) compared to that observed in patients with remission at baseline (W0_Rm). This module consisted of several genes (K00677, K02536, K03269, K00748, K02560, etc.), and the expression levels of most of these genes were significantly decreased in W4_Rm compared to W0_Rm and W4_NRm (Fig. 6D). Notably, the abundance of species, including Escherichia coli, Klebsiella pneumoniae, and Haemophilus parainfluenzae, which were stratified to encode these genes by Humann3, was decreased in the W4_Rm group (Fig. 4A and 6D). These results suggested that the depletion of species such as E. coli and Klebsiella after FMT might reduce LPS production, which might result in better therapeutic effects in the remission group. Moreover, the concentration of indolelactic acid was higher in W4_Rm than in W0_Rm and W4_NRm (Fig. 6E), which was found to be consistent with the higher abundance of the tryptophanase gene (K01167) and the taxa *Alistipes* sp. strain 3BBH6 and Odoribacter splanchnicus, which converted tryptophan to indolelactic acid.

## DISCUSSION

In this study, we explored the clinical outcomes of capsulized FMT treatment in patients with active UC. We also performed a general analysis of the gut microbiota and serum metabolites to identify specific taxonomic, functional, and metabolite changes associated with clinical outcomes after FMT. Capsulized FMT could induce clinical remission in 57.1% (12 of 21) of patients with UC and clinical response in 76.2% (16 of 21) of patients at week 12. The gut microbiome and serum metabolites in samples obtained from patients were significantly altered after capsulized FMT treatment, especially at week 4. Improvements in microbial richness, depletion of opportunistic pathogens (Escherichia coli, Enterobacter hormaechei, and Salmonella enterica), pathways such as LPS biosynthesis, and proinflammatory metabolites (12,13-DiHOME) and enrichment of beneficial microbes (Faecalibacterium prausnitzii and *Alistipes* sp.), pathways such as acetyl-CoA biosynthesis, and anti-inflammatory metabolites (indolelactic acid), and their interactions were identified to be associated with the therapeutic effects of FMT.

The key features distinguishing this study from previous studies on FMT in UC patients were the low intensity of microbiota transplantation using capsules and the high efficiency of clinical remission. Previously, colonic transendoscopic tubes or enemas were the main methods used to deliver fecal microbiota transplantation, and the treatment cycle was relatively long. For example, Paramsothy et al. first achieved a single colonoscopic delivery of FMT to the right colon and then performed enemas 5 days per week for 8 weeks (9). Samuel et al. demonstrated the efficacy of FMT by performing enemas twice a week (6), and Zhang et al. used a single fresh FMT through the midgut (17). Pai et al. reported on a colonoscopic infusion at the baseline, followed by the oral administration of capsules two times per week for 6 weeks in patients between the ages of 3 and 17 years (18). All invasive operations were risky and resulted in increased patient costs (19). In our study, all participants received three capsulized FMT procedures within a week and none exhibited serious side effects. The results showed that the abdominal pain score, diarrhea score, bloody stool score, intestinal mucosal lesions, and total Mayo score of patients decreased significantly after FMT. This modality was more acceptable than colonoscopy or enemas for patients. Notably, the clinical remission rate (57%) was higher than the values reported in previous quasiexperimental or randomized controlled trial (RCT) studies (37.0%; 95% confidence interval [CI], [28.8 to 45.9]) and higher than that in studies using capsules (20). In an open-label pilot study (13), seven patients with active UC showed temporary improvement in clinical symptoms after the daily administration of FMT capsules for 50 days. There was no change in the α diversity of the bacterial community in the fecal samples (13). In another randomized pilot study on encapsulated oral FMT for long-term maintenance therapy (16), 2 of 6 (33.3%) patients with active UC achieved clinical remission after 12 weeks of daily oral administration of encapsulated FMT. On the other hand, Arndt et al. reported that when patients with active UC received capsulized FMT every day for 5 consecutive days during 12 weeks, 2 of 10 patients exhibited worsened colitis, and 7 of 8 patients exhibited a partial improvement in the Mayo score (5.8 ± 1.7 to 2.6 ± 1.1) (10). Thus, capsulized FMT might be an alternative in treating patients with UC to induce remission. However, the issues related to the preservation of capsules at home must be addressed.

Generally, if clinical symptoms were relieved 4 weeks after FMT, most patients could be in a stationary phase. The longer the time required for symptom remission, the worse the prognosis. We attempted to reveal the relationship between lesion regions (E1, E2, and E3) or disease severity and the clinical response, but there were no significant differences in the efficacy of capsulized FMT, indicating that the impact of capsulized FMT was unrelated to the lesion area and disease severity. We also observed remarkable and specific differences in gut microbiota and metabolites between the Rm and NRm groups at week 4 after FMT. Notably, previous studies also investigated changes in the microbiota at 4 weeks after FMT, and results show consistent trends (6, 9, 21). We might assess whether patients would benefit from FMT at 4 weeks after FMT. If not, another therapy must be provided to such patients as soon as possible.

We also observed that microbial richness was significantly decreased in patients with UC compared to that in healthy individuals. However, microbial richness levels were improved to levels comparable to those of donors only in patients who achieved clinical remission at week 12 after FMT. Improvement of gut microbial richness after FMT has been reported in previous clinical studies on UC patients (6, 9, 22) and was suggested as an important indication for the successful treatment of patients with FMT (6, 23–25). Moreover, the higher gut microbial richness of donors was reported to be associated with remission in UC patients (26). Overall, the bacterial community structure shifted to be closer to donor samples after FMT. Specifically, levels of *Prevotella_9* were notably increased and became equal to those of the dominant genus after FMT in our 16S rRNA gene data set, accompanied by decreased levels of *Bacteroides*. Since both of these genera belong to *Bacteroidales*, it is unclear why this replacement occurred (27), but the enrichment of *Preovella_9* after FMT was frequently reported in other studies on FMT treatment (9, 28). Other important changes after FMT in the bacterial profile included the eradication of *Veillonella* and *Enterococcus*, which are well-known opportunistic pathogens, and the enrichment of *Alloprevotella* and *Odoribacter*, which can produce short-chain fatty acids (SCFAs) (29, 30) that benefit human health.

Microbes and functional microbial pathways associated with clinical remission were further analyzed based on 16S rRNA gene and metagenomic data sets. Compared with the baseline, the increased relative abundance of species, including *Faecalibacterim* (*F. prausnitzii*), *Butyricimonas*, *Alistipes* (*Alistipes* sp. strains 3BBH6, 5CBH24, and 5CPEGH6 and *A. shahii*), *O. splanchnicus*, and *Christensenellaceae_R-7*, was uniquely associated with clinical remission after FMT. Among these, *Faecalibacterim*, *Butyricimonas*, and *Alistipes* are well-known SCFA-producing bacteria (29) and anti-inflammatory commensal bacteria that play a role in IBD (31), obesity (32), appendectomy (33), and depression (34). Interestingly, a recently published clinical study reported that *O. splanchnicus* was a key strain that could promote both metabolism (producing SCFAs) and mucosal immunity (inducing regulatory T cells) and has protective effects against ulcerative colitis (35). The family *Christensenellaceae*, reported to be widespread and associated with human health, was depleted in individuals with CD, UC, and IBS (36). A decrease in specific taxa, including Escherichia*_Shigella* (E. coli), Enterobacter (*E. hormaechei*), and Citrobacter freundii, was also exclusively linked with clinical remission; this was consistent with the results of previous reports, which showed that they acted as pathogens that caused IBD, diarrhea, and other infections (37–39). Microbial functions that were specifically improved included acetoacetate plus acetyl-CoA, gluconeogenesis to fructose-6P, and thiamine formation. Importantly, the relative levels of biosynthesis of the lipopolysaccharide KDO2-lipid A and lipid IVA and the extent of activation of superpathways for *N*-acetylneuraminate degradation were exclusively decreased during clinical remission after FMT, which coincided with changes in the levels of Escherichia coli, Klebsiella, and Salmonella enterica, which contribute to the formation of LPS (40, 41). As a major endotoxin that strongly stimulates host innate immunity, LPS derived from enteric opportunistic bacteria has been implicated in IBD, autoimmune diseases, and metabolic disorders (42, 43). Therefore, the prevention of LPS accumulation provides protection against the development of severe colitis (41).

Serum metabolite profiles were significantly different between the baseline and week 4 after FMT. Among these profiles, elevated levels of indolelactic acid, PGH2-EA, and isohyodeoxycholic acid were associated with a favorable treatment output, while increased levels of 12,13-DiHOME were associated with worse outcomes. PGH2-EA, a prostaglandin, showed anti-inflammatory properties in ulcerative colitis (44). The 12,13-diHOME produced by bacterial epoxide hydrolase genes altered the expression of PPARγ (peroxisome proliferator-activated receptor γ)-regulated genes, reduced anti-inflammatory cytokine secretion and the number of regulatory T cells, increased inflammation, impeded immune tolerance and was involved in the development of atopy, eczema, or asthma (45). The elevation in the levels of tryptophan biosynthesis because of FMT might be another important factor contributing to therapeutic success. Tryptophan was further metabolized by gut microbes into indolelactic acid, indole acetic acid, and indoxyl sulfuric acid in patients achieving clinical remission (46). Metabolites could act as signals that activate the innate immunity of intestinal mucosa and induce a rapid inflammatory response (47). Indolelactic acid modulated *ex vivo* immune responses of human CD4^+^ T cells and monocytes in a dose-dependent manner by acting as an agonist of both the AhR and hydroxycarboxylic acid receptor 3 (48). In fact, we made an effort to do some investigation in order to clearly show the important connection between the microbiota or metabolites and the clinical response. The findings demonstrated that plasma levels of tumor necrosis factor alpha (TNF-α), interleukin-17 (IL-17), IL-10, and transforming growth factor beta (TGF-β) did not significantly change, nor did immune cells in the peripheral blood (data not shown). Nevertheless, we discovered that indolelactic acid might reduce intestinal inflammation in colitis brought on by dextran sulfate solution (DSS) (data not shown). More research must be done on the underlying mechanisms of indolelactic acid or other colitis in colitis.

In general, we demonstrated the efficacy of FMT in patients with mild to severe UC through well-prepared donor stool capsules administered orally three times a week. Capsulized FMT could accelerate the metabolism of tryptophan by increasing the abundance of *Alipipes* sp. strain 3BBH6, Odoribacter splanchnicus, and other bacteria, and enable the production of indole lactic acid and other products that alleviate intestinal inflammation.

## MATERIALS AND METHODS

### Study design.

We conducted a single-arm pilot clinical trial by enrolling 22 patients with active UC between June 2016 and June 2019 at Zhongshan Hospital in Xiamen University. Nine donors were included in the study, and their stool was extracted and filled into capsules. To increase microbial diversity, every patient randomly received FMT infusions from two or three donors. Patients underwent three capsulized FMT procedures (one every 2 days) within a week. After 12 weeks of follow-up visits, clinicians assessed the effect of FMT on UC patients. Maintenance therapy involved the administration of a stable dose (prior to enrollment) of 5-ASA/SASP (salicylazosulfapyridine) and prednisone that was mandatorily tapered to 5 mg (>10 mg/day) or 2.5 mg (≤10 mg/day) per week. Stool samples of patients with UC were collected at weeks 0, 1, 4, and 12 for microbiome analysis, and blood samples were collected for laboratory examination and metabolomic analysis. This study was approved by the Zhongshan Hospital of Xiamen University and was registered at ClinicalTrials.gov (registration no. NCT03426683).

### Donor management.

Donors were recruited via an advertisement at Xiamen University, answered a questionnaire during the screening process, and replied to questions regarding their lifestyle and medical history in an interview. Potential donors sequentially underwent medical examinations to ensure that there was no risk of an infection. Eligible donors underwent medical examinations every 3 months and were instructed to maintain healthy eating habits. The following tests were conducted using serum samples: blood routine, urine routine, liver function, renal function, C-reactive protein (CRP), erythrocyte sedimentation rate (ESR), HIV, hepatitis A, B, and C, tumor markers, TORCH (antibodies against toxoplasmosis, rubella, cytomegalovirus, herpes simplex, and HIV), Epstein-Barr virus (IgG and IgM), cytomegalovirus (IgG and IgM), and H. pylori (IgG) tests. The fecal occult blood test, routine enteric pathogen test, and C. difficile toxin B test were conducted using fecal samples. The criteria for the exclusion of the donors were as follows: a personal history of any infection, such as hepatitis or HIV; personal history of cigarette smoking or drinking; drug use in the previous month, including antibiotics, probiotics, or probiotics; gastrointestinal diseases, such as functional gastrointestinal disorders, infections, and polyps; autoimmune disease; metabolic syndrome; and other diseases.

**(i) Inclusion criteria.** Male and female patients were included if their age was between 18 and 70 years and if they had active ulcerative colitis and had a poor response to drugs (including 5-aminosalicylic acid, salicylazosulfapyridine, and prednisone) (total Mayo score of 4 to 12). The total Mayo score, a component representative of the clinical symptoms (stool frequency and rectal bleeding), endoscopic assessment, and physician rating, ranged from 0 to 12.

**(ii) Exclusion criteria.** We excluded patients with contraindications for gastrointestinal endoscopy, patients with other serious diseases, such as respiratory failure, heart failure, and severe immunodeficiency, patients with indication of surgery or previous colonic surgery, patients with a gastrointestinal infection, patients using antibiotics or probiotics 1 month before enrollment, patients who were pregnant or preparing for pregnancy, and patients who had a history of other diseases affecting the gut microbiota, such as metabolic syndromes and autoimmune diseases.

### Withdrawal criteria.

Patients could withdraw from the study at any time. To ensure the reliability of data, patients were informed that they needed to contact clinicians before withdrawal and provide reasons for leaving.

### Preparation of capsules.

Fresh stool was collected from donors in a sterile plastic container. The dry weight of the stool needed to be more than 100 g. Fresh fecal samples (25%) were mixed with saline (60%) and pharmaceutical-grade glycerol (15%) in a semiautomatic extraction instrument (Treatgut TG-01; Anjiezhishan Company, China). The instrument was launched to start the process of gradual separation. After the extraction process was performed using sterile tubes, the supernatant was collected and centrifuged to obtain the precipitate containing fecal microbiota. The precipitate was mixed thoroughly, and the capsules (DrCaps; 19504907) were filled. Each capsule contained an average of 0.9 g of precipitate of fecal microbiota and was immediately frozen at −80°C.

### Interventions.

We obtained informed consent from patients prior to their inclusion in this study. Authorized physicians administered FMT under monitored clinical settings. Before taking capsules orally, patients were required to fast overnight for at least 8 h. All patients received FMT in the morning. Capsules stored at −80°C were returned to room temperature before they were administered orally. Patients took the capsules with warm saline. Patients were kept under observation for 6 h after receiving FMT in the hospital to determine any adverse effects. Capsulized FMT was administered in three separate procedures (30 capsules every 2 days) within 1 week, and follow-up was performed for up to 12 weeks. The total weight of stool administered over the three FMT procedures was about 90 g. Stool samples of patients were collected for microbiome analysis at weeks 0, 1, 4, and 12, and blood samples of patients were collected for laboratory examination and metabolomic analysis. A colonoscopy assessment was conducted at the baseline (week 0, before FMT) and week 12. The time points for weekly assessment were W0 (baseline, week 0), W1 (week 1), W4 (week 4), and W12 (week 12).

### Medication during enrollment.

UC maintenance therapy was provided to patients during FMT. Because patients had a poor response to mesalazine, SASP, or prednisone, stable doses of drugs were used during follow-up after FMT. A subject treated with 5-ASA or SASP continued to receive the same dose of drugs administered during the study. A subject who was treated with prednisone would continue to receive it after a mandatory tapering of the dose: prednisone at >10 mg/day had a mandatory taper of 5 mg per week until the administration of 10 mg/day, and prednisone at ≤10 mg/day had a mandatory taper of 2.5 mg per week.

### Outcomes.

Clinical response at week 12 was defined as a reduction of ≥3 points in the total Mayo score and a decrease of ≥30% from the baseline score (49, 50). Clinical remission was defined as a total Mayo score of ≤2 at week 12. Endoscopic remission should result in mucosal healing and a Mayo score of ≤1 (50).

### The 16S rRNA gene in fecal microbiota and shotgun metagenomic sequencing.

Fresh fecal samples were collected and frozen at −80°C until DNA extraction. According to the manufacturer's protocol, total fecal DNA was extracted from 0.25 g of each homogenized sample using the QIAamp PowerFecal DNA kit (Qiagen). The DNA yield and quality were then checked with a Multiskan GO spectrophotometer (Thermo Fisher Scientific, USA). To identify bacterial communities, 16S rRNA gene V3-V4 amplicon libraries were generated in a 20-μL reaction mixture containing 10 μL KAPA HiFi HotStart ReadyMix (KAPA Biosystems, USA), 2 μL DNA (60 ng), and 1 μL of each of the barcoded primers (10 μM). The forward and reverse primers used were 5′-CCTACGGGNBGCASCAG-3′ and 5′-GGACTACNVGGGTWTCTAAT-3′, respectively. The PCR cycle conditions were as follows: 95°C for 3 min, followed by 30 cycles at 95°C for 20 s, 60°C for 30 s, 72°C for 30 s, and finally 72°C for 10 min. In accordance with the protocol of the AxyPrep PCR cleanup kit (Axygen, USA), we purified the PCR product of each sample and assessed their concentrations using Qubit 3.0 (Thermo Fisher Scientific, USA). To perform shotgun metagenomic analysis, 100 ng DNA of each sample was cut into strands with a length of 350 bp using the Bioruptor NGS sonicator (Diagenode). This was followed by steps such as end modification, A-tail addition, sequencing adapter addition, purification, and enrichment, in accordance with the protocol for the Illumina NEBNext Ultra II DNA library prep kit. The insert size of the library was detected using Agilent 2100 (Agilent, USA). Finally, 16S rRNA gene and metagenomic libraries were sequenced on the HiSeq 2500 platform (Illumina, San Diego, CA, USA) with 250-bp paired-end sequencing reagents and Novaseq 6000 (Illumina, Inc., San Diego, CA, USA) with 150-bp paired-end sequencing reagents, respectively, at Xiamen Treatgut Biotechnology Co., Ltd. Raw sequences were deposited in the NCBI Sequence Read Archive.

### Microbial bioinformatic analyses.

Raw V3-V4 sequencing reads were assembled to generate high-quality reads using FLASH (51) with parameters such as -M 200 and -x 0.15. Primers were further removed using Cutadapt, and the generated reads were chimera checked and clustered to generate OTUs exhibiting 97% similarity using USEARCH (52). To perform the taxonomic classification of bacteria, representative sequences of OTUs were classified against the Silva database (53) using the RDP Classifier at a confidence level of 50% (54). The OTU table was resampled, and 19,295 reads/samples were selected for downstream analyses. Raw shotgun sequencing reads were filtered using Trimmomatic (55) to remove sequencing adaptors and low-quality and low-complexity reads. Kneaddata was used (https://huttenhower.sph.harvard.edu/kneaddata) to remove contaminants from the human host. The remainder of the reads were classified using Kraken2 (56) to determine the taxonomic composition and analyzed using Humann3 (57) to perform functional annotation.

### Serum sample collection and metabolomics.

Serum samples were collected and immediately frozen at −80°C until metabolite extraction. Total metabolite content was extracted from 0.05 g of each of the serum samples, followed by liquid chromatography-tandem mass spectrometry (LC-MS/MS) analyses, performed using the ultrahigh-performance liquid chromatography (UHPLC) system (1290, Agilent Technologies) with an ultraperformance (UPLC) HSS T3 column (2.1 mm by 100 mm, 1.8 μm) coupled to the Q Exactive system (Orbitrap MS; Thermo). MS raw data were filtered if the following criteria were met: (i) metabolites present in less than 50% of all samples in a group were removed, and (ii) missing values were replaced by half of the minimum values found by default in the data set. Peaks were annotated against an in-house MS/MS database constructed using HMDB, Metlin, and Mona. Finally, the relative content of each peak was determined by the normalization method of peak area to generate percentage value for each metabolite.

### Statistical analyses and visualization.

Microbial α-diversity indexes, including richness (Observed and Chao1), Shannon diversity (Shannon), and Pielou's evenness (Evenness), were computed using the vegan package (58). Microbial β diversity was assessed based on the Euclidean distance, and significance was tested using permutational multivariate analysis of variance (PERMANOVA) with 9,999 permutations using adonis in the vegan package. Metabolomic profiles were analyzed via partial least-squares-discriminant analysis (PLS_DA) using the mixOmics package. The significance of the diversity indexes, individual taxa, and metabolites among groups was determined using a nonparametric Kruskal-Wallis rank sum test and Benjamini-Hochberg corrections with the Agricolae package (59), while the significance of Mayo score between groups before and after FMT was determined by paired Wilcoxon rank sum test. Effect size analysis was performed according to the method described by Wang et al. (60). Pearson correlations were calculated between species and metabolites, species and MetaCyc pathways, species and KEEG modules, metabolites and KEGG modules, and metabolites and MetaCyc pathways. Finally, results were visualized using the custom R script, mainly based on ggplot2 from the R package (61) or VennDiagram (62). These analyses were performed using R v.3.3.2 (63).

### Data availability.

The accession numbers of raw sequences have been deposited in the NCBI Sequence Read Archive under BioProject accession no. PRJNA672846.
