# Supplementary material for: Capsulized Fecal Microbiota Transplantation Induces Remission in Patients with Ulcerative Colitis by Gut Microbial Colonization and Metabolite Regulation
Source: Microbiol Spectr. 2023 Apr 24;11(3):e04152-22. doi: 10.1128/spectrum.04152-22 (PMC10269780; doi:10.1128/spectrum.04152-22)
Supplement: Supplemental file 1 — Fig. S1 to S10. Download spectrum.04152-22-s0001.pdf, PDF file, 3.5 MB [file spectrum.04152-22-s0001.pdf]

Supplementary Figures

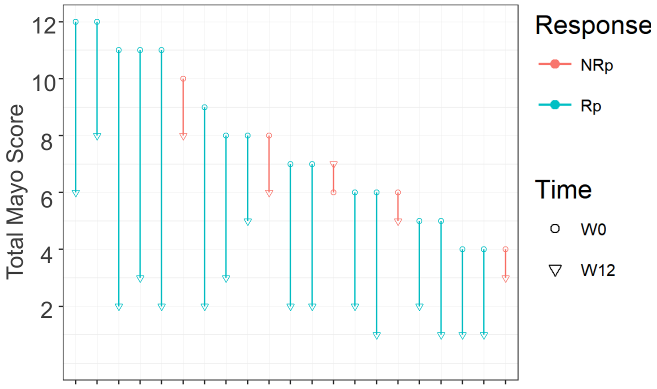

Fig S1 Effect of fecal microbiota transplantation on 12-week response in patients with ulcerative colitis. Mayo scores for UC patients before and after treatment with FMT. The parallel line shows the change in Mayo score for individual patients, with a solid line indicative of values for FMT. Each line starts at the baseline (W0, circle) and finishes at its endpoint (W12 in triangle for FMT), while the achievement of clinical remission is indicated in green and non-clinical remission is indicated in red. Stars in boxplots present the mean Mayo scores of each group. FMT indicates oral capsulized fecal microbiota transplantation

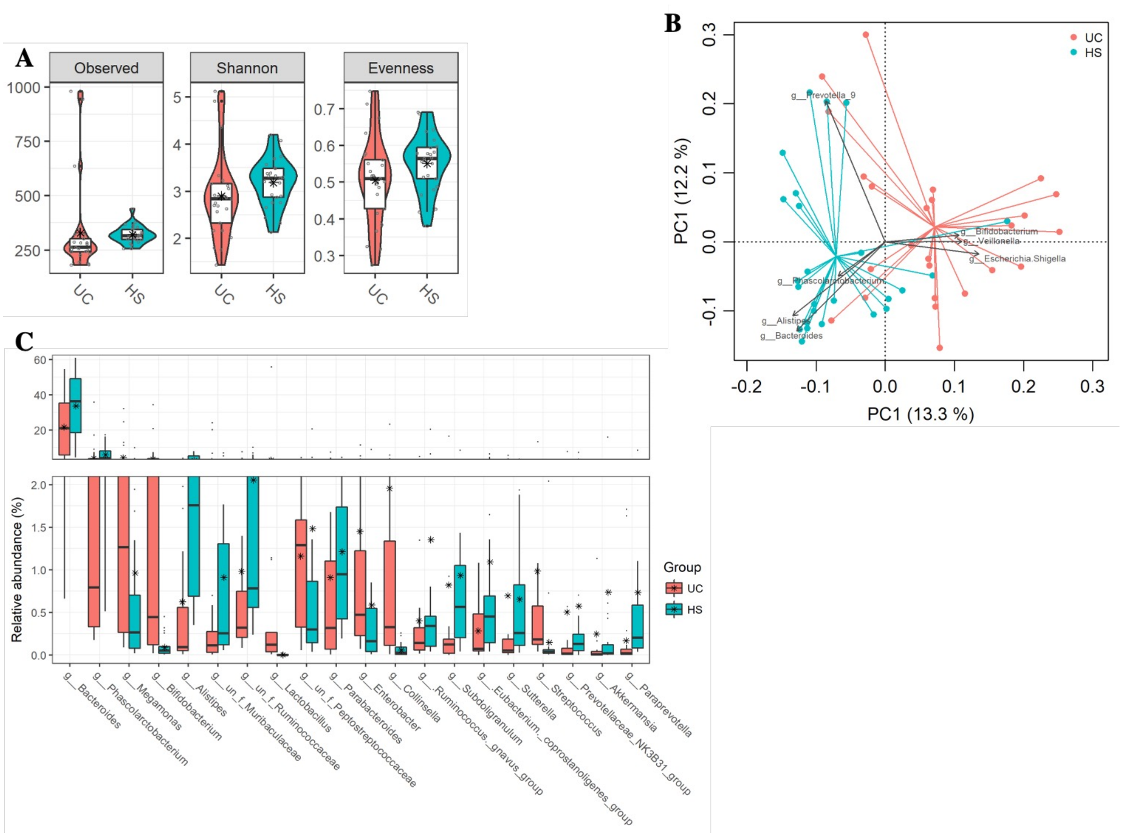

Fig S2 Differences in gut bacterial communities between UC and healthy subjects (HS), based on 16S V3-V4 sequencing data. (A) Alpha-diversity indexes of UC and HS. (B) Differences in gut bacterial communities, assessed by principal component analysis of the Euclidean distance. The top genera were fitted to PCA with a significant level. (C) A significant difference in relative abundances of the top 25 genera in samples from patients and donors. UC indicates ulcerative colitis. HS indicates the health.

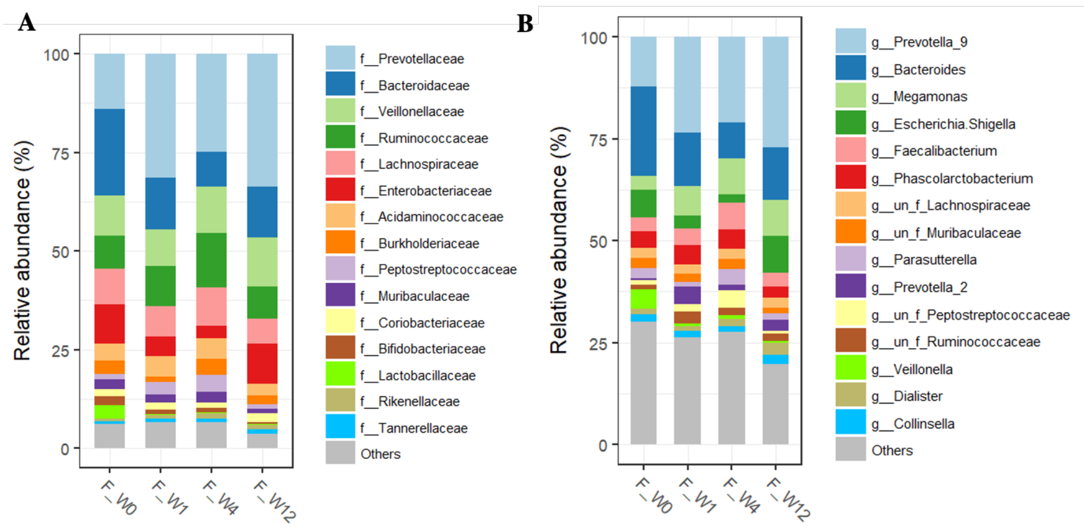

Fig S3 Differences in gut bacterial communities after treatment with FMT in different times. (A) Relative abundances of the top family in samples from patients treated with FMT. (B) Relative abundances of the top genera in samples from patients treated with FMT.

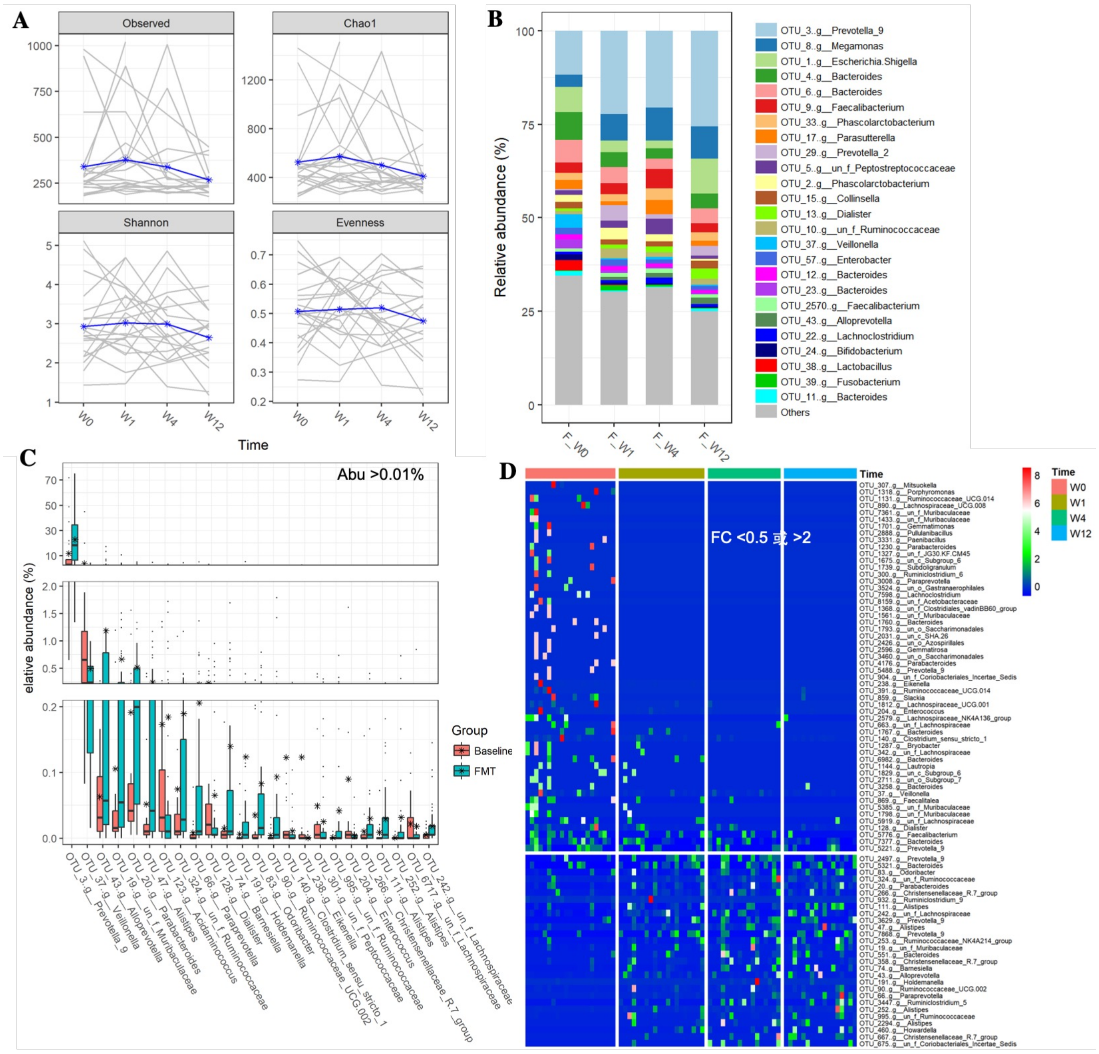

Fig S4 Differences in gut bacterial communities after treatment with FMT in different times on OUT level. (A) Changes of alpha-diversity indexes estimated by richness (Observed OTUs, Chao1), Shannon, and Pielou's evenness of each individual over time. The star indicate the mean value of each timepoint. (B) Relative abundances of the top 25 OTUs in samples from patients treated with FMT. (C) The predominant OTUs (relative abundance > 0.01%) that were significantly (P<0.05) differentiated between baseline and after FMT. The star indicate the mean value of the group. (D) Heatmap of OTUs that were significantly (P<0.05) differentiated between baseline and after FMT and possessed FC>2 or FC<0.5 in samples from patients treated with FMT.

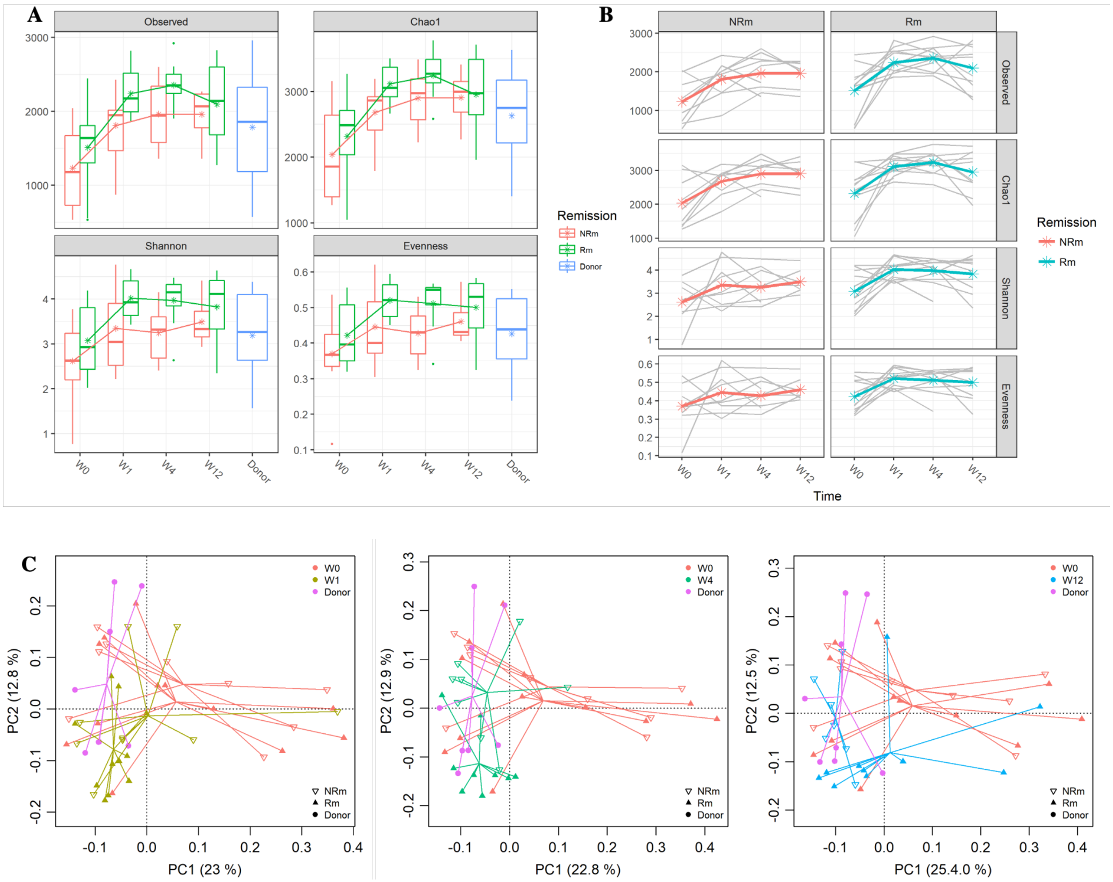

Fig S5 Changes in gut microbial communities (metagenomics) associated with Rm and NRm after FMT. Alpha-diversity of Rm, NRm, and donor samples, estimated by richness (Observed species, Chao1), Shannon, and Pielou's evenness at each timepoint (A) and of each individual over time (B) . The black line indicates significant ($P<0.05$) differences between the two groups covered by the line. (C) Differences in gut microbial community structures of Rm, NRm, and donors between the baseline and the other time point during follow-up after FMT, by PCA based on Euclidean distance of species abundances. Data were Hellinger-transformed. More significance test results were recorded in Table S2.

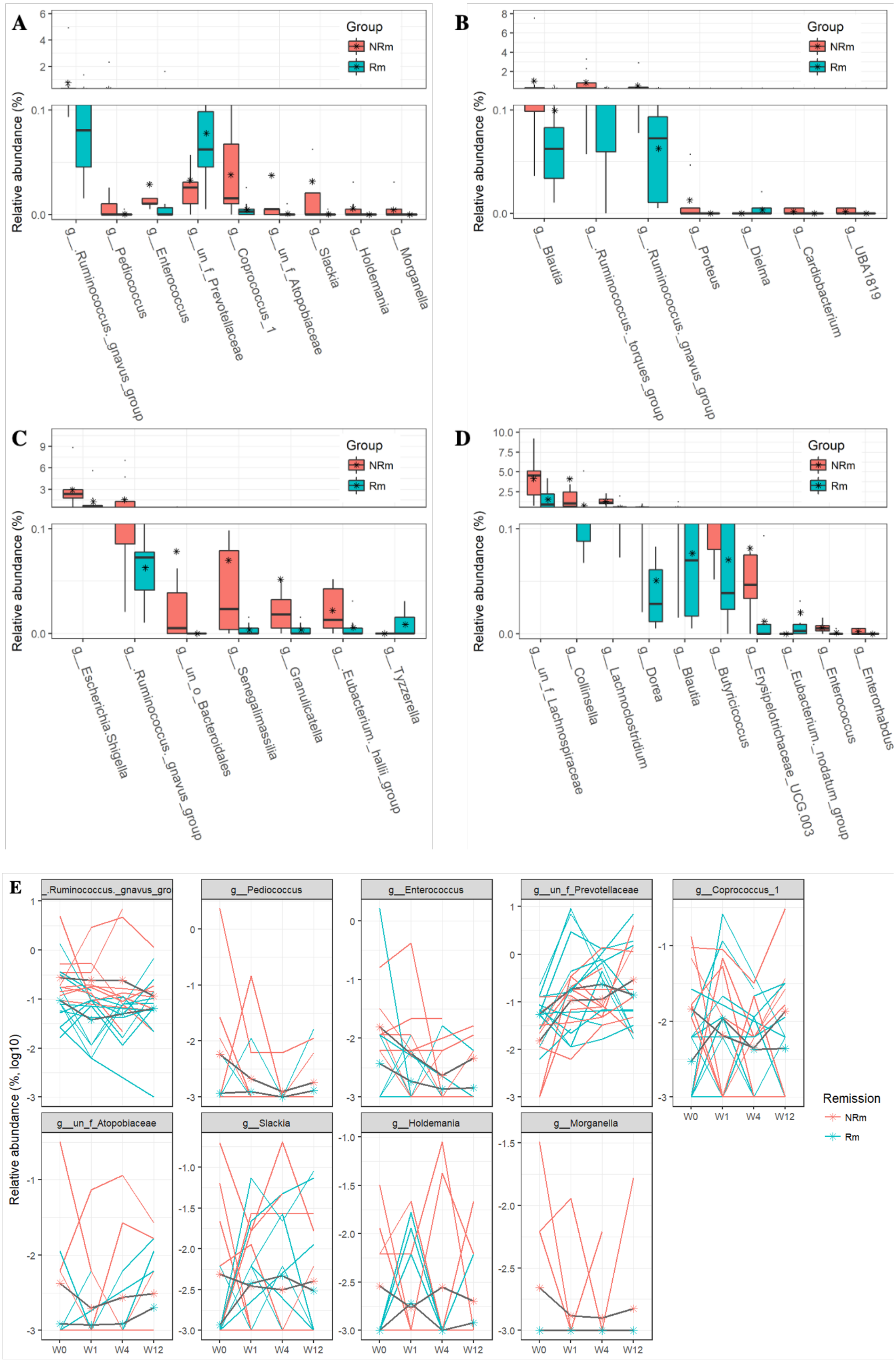

Fig S6 Relative abundances of the bacterial genera significantly different between Rm and NRm at W0 (A), W1(B), W4 (C), W12 (D). (E) Relative abundances of the bacterial genera significantly different between W0_Rm and W0_NRm over time.

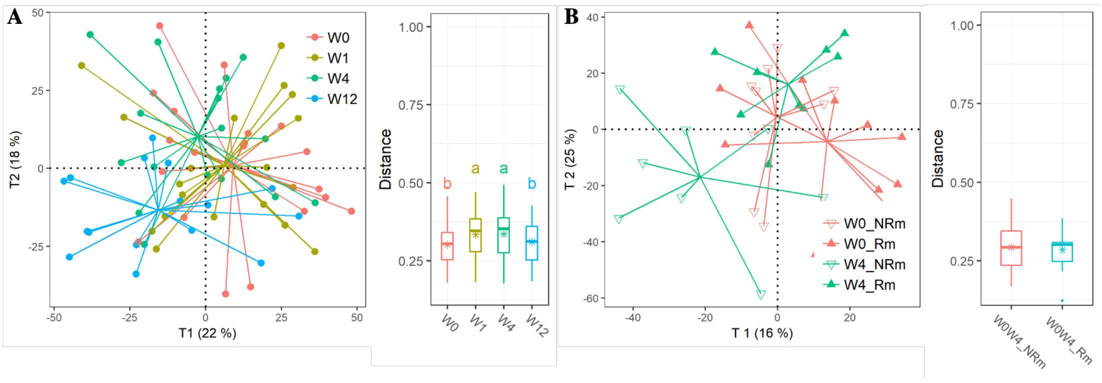

Fig S7 Metabolic profiles in negative ion model associated with clinical remission. (A) PLS_DA analysis of metabolomic profiles by time points (left panel) and Euclidean distances of samples within the same time points (right panel). (B) PLS_DA analysis of metabolomic profiles at Week 0 and Week 4 after FMT with achieving clinical remission or not achieving clinical remission (left panel), as well as Euclidean distances of patients between Week 0 and Week 4 (right panel).

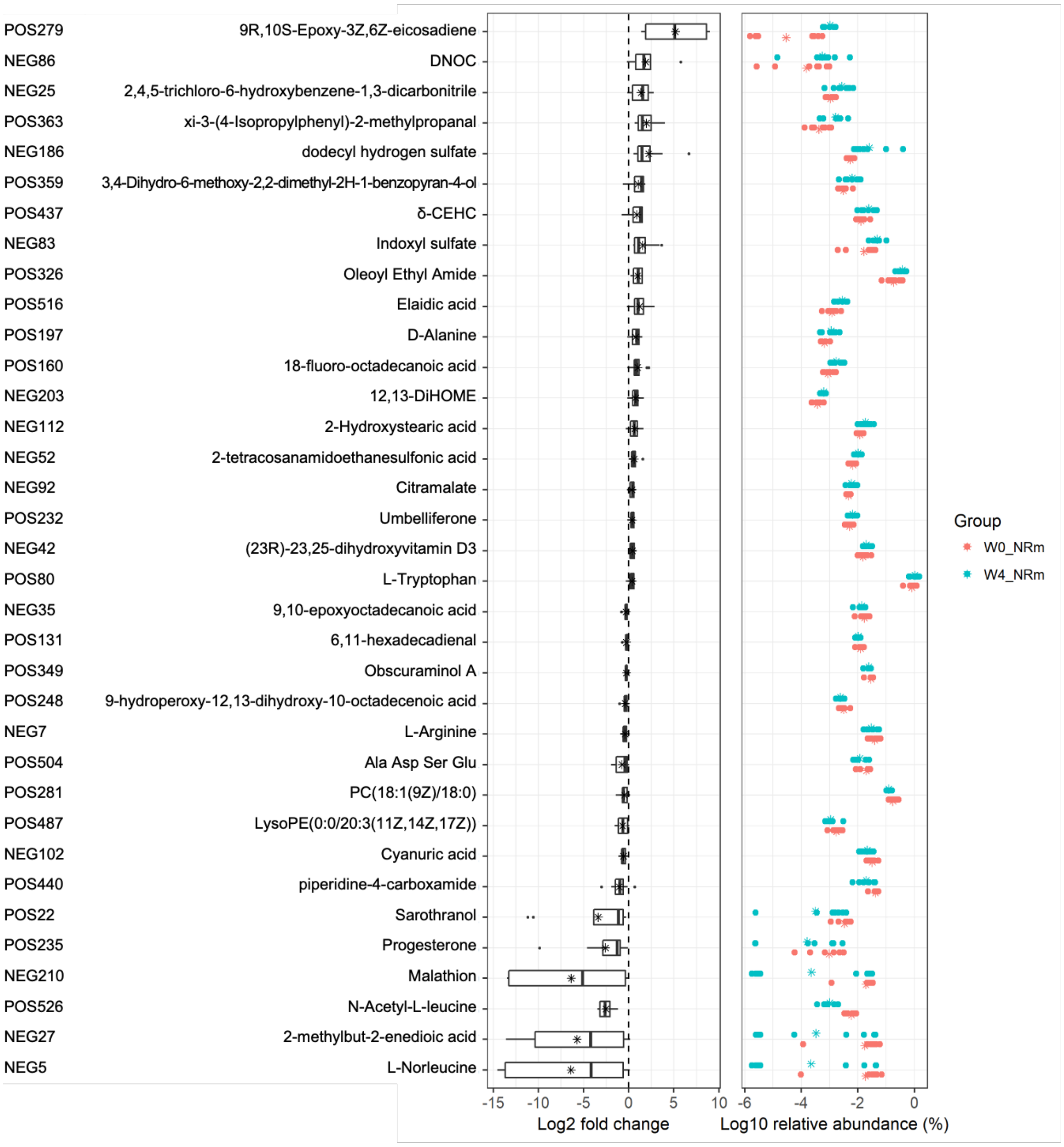

Fig S8 Metabolites that were significantly decreased or increased uniquely in W4_NRm compared with W0_NRm group (namely, not significantly differentiated in W4_Rm vs W0_Rm), were filtered by VIP score >1, and were structurally identified in databases.

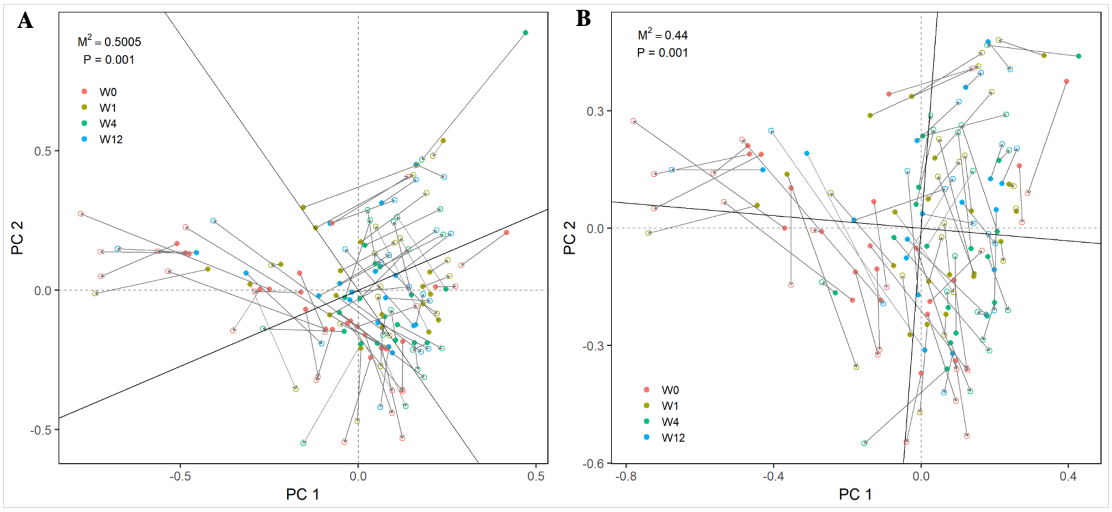

Fig S9 Inter-omic correlations between gut microbial species and functional features, by Procrustes analysis of gut microbial species and KEGG modules (A) and MetaCyc pathways (B).

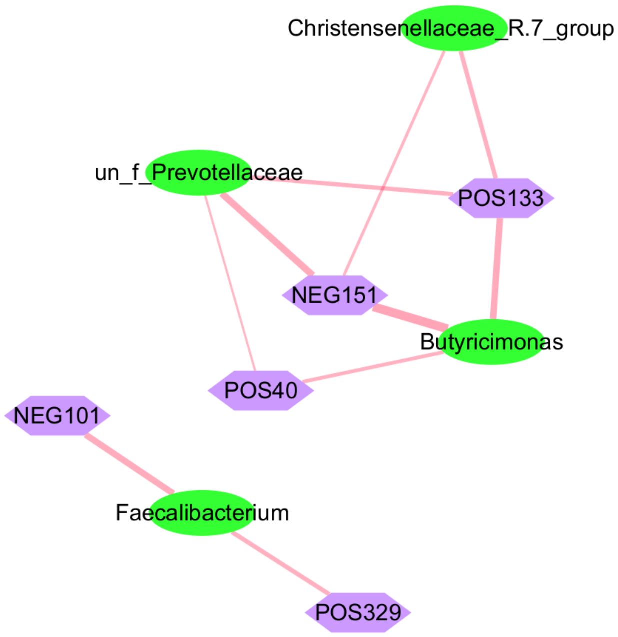

Fig S10 Correlations between gut microbial species from 16S V4 and metabolites.
